# Historical and Biographical Address, Delivered before the Cortland County Medical Society, N. Y., at the Fiftieth Anniversary Meeting, Aug. 10th, 1858

**Published:** 1860-06

**Authors:** 


Historical and Biographical Address: Delivered before the Cortland County Medical Society, N. Y., at the Fiftieth Anniversary Meeting, Aug. 10th, 1858. By George W. Bradford, M. D., Secretary of the Society.

We are much obliged to our esteemed friend, Dr. Bradford, for a copy of the above address. It contains a brief history of the formation of the Cortland County Medical Society in 1808 —a sketch of each of its original founders—and a full list of the members up to the present time. It is full of interesting incidents, and cannot fail to possess much local interest and value.

